# Hierarchy: from nature to artificial

**DOI:** 10.1093/nsr/nwaa245

**Published:** 2020-10-08

**Authors:** Bao-Lian Su, Dongyuan Zhao

**Affiliations:** State Key Laboratory of Advanced Technology for Materials Synthesis and Processing, Wuhan University of Technology, China; Laboratory of Inorganic Materials Chemistry (CMI), University of Namur, Belgium; Department of Chemistry, State Key Laboratory of Molecular Engineering of Polymers, Shanghai Key Laboratory of Molecular Catalysis and Innovative Materials, Laboratory of Advanced Materials, and iChEM, Fudan University, China

Hierarchy is present everywhere in our planet, from family, society, urban construction, ecological systems to living organisms, although usually we neglect it. In the biosystem, to achieve transfer, exchange and transformation of substances and energy with extremely high efficiency and minimized energy consumption, evolution by natural selection has endowed many classes of organisms with hierarchically porous networks. Building hierarchy at multiple length periods and scales is a common strategy in nature to obtain living matter with extraordinary and unusual sets of properties that are highly desirable in a wide range of artificial materials.

Saving energy and raw materials, and protecting our world with the concurrent need to improve human life for future generations lead us to make intensive efforts in the rational design of hierarchically porous systems for a maximized efficiency of each specific application. We will undergo not only an industrial revolution, but also a progress of our society.

Over the last decade, tremendous research has been focused on the synthesis and applications of hierarchically porous materials. This subject became a hot topic and will continue to be prosperous in the years to come. A large series of domains, from biotechnology, biomedicine, (photo)catalysis, energy conversion and storage, optics, gas sensing and separation processes to the encapsulation of biomolecules and bio-organisms, have a fervent interest in hierarchically porous materials.

This special topic aims at highlighting the state-of-the-art progresses in this hot field and includes one research highlight, four perspectives, three research articles, four reviews and one interview. First, Sanchez highlights some of the most significant research of the last decade. In their perspective, Chen *et al*. show a detailed introduction about hierarchy concept and its role for maximized efficiency in society, ecosystems and life. Zhou *et al*. reveal the importance of the surface diffusion in hierarchical/nano-zeolites. Yue *et al*. present green fabrication of hierarchical zeolite from natural minerals. Kim *et al*. demonstrate their new invention and perspective for energy storage by spinodal decomposition.

In their research article, Wei *et al*., inspired by nature, illustrate their new concept of temporally–spatially sequential harvesting of solar light in one hollow multishelled structured particle. Tian *et al*., by mimicking cells, construct hierarchically hollow-structured submicrometer photoreactors. Lu *et al*. present a new route to prepare low-valence metal oxide-based hierarchically porous monoliths.

Four reviews survey the synthesis and applications of a large panel of hierarchically porous materials covering metal oxides, carbon, zeolites and metal–organic frameworks (MOFs). Wu *et al*. review the most significant preparation methods and the effect of different organizational, structural and geometric parameters of porous hierarchy on their electrochemical behavior. Li *et al*. summarize hierarchically mesoporous TiO_2_ materials and their applications in energy storage and environmental protection. Peng and coworkers focus on the diffusion and catalyst efficiency of hierarchical zeolites and industrial catalysts. Feng *et al*. overview recent advances in the methodology of hierarchically porous MOF synthesis and their applications.

In the interview, Prof. He, the pioneer of the green carbon science, highlights future prospects of hierarchically porous materials and their importance in the development of new industrial processes for the society.

We thank all the authors and editorial staff for their efforts to make this theme possible. A special thank to Prof. Mingyuan He and Dr Li-Hua Chen for the interview. We also thank Prof. Sanchez for his important effort to point out the recent milestones achieved in the field. Beyond it, one can see the world differently, hierarchically since every system of organization applied to the world is arranged hierarchically, and one can clearly realize the universal principle of hierarchy around world everywhere. ‘Do as Nature, Work as Nature and Produce as Nature’ should be the essence of new synthetic strategies of hierarchically porous materials and for their extensive use in our daily life, in regenerative medicine and in industry.

